# The PML-RARA fusion is not detectable in historical blood samples of acute promyelocytic leukaemia patients

**DOI:** 10.1007/s00277-021-04472-5

**Published:** 2021-03-01

**Authors:** William G. Dunn, Muxin S. Gu, Margarete A. Fabre, Jonathan Cooper, Josep F. Nomdedeu, Laura Koumas, Katerina Nicolaou, Jiangxiang Chi, Paul Costeas, George S. Vassiliou

**Affiliations:** 1 Wellcome-MRC Cambridge Stem Cell Institute, Jeffrey Cheah Biomedical Centre, Puddicombe Way, Cambridge, CB2 0AW UK; 2 Department of Haematology, Cambridge University Hospitals NHS Trust, Cambridge, UK; 3 Wellcome Sanger Institute, Wellcome Genome Campus, Hinxton, Cambridge, CB10 1SA UK; 4 Department of Haematology, Hospital de la Santa Creu I Sant Pau, Barcelona, Spain; 5 Center for the Study of Haematological Malignancies, Karaiskakio Foundation, Nicosia, Cyprus; 6 Cyprus Cancer Research Institute, Nicosia, Cyprus

Dear Editor,

Acute promyelocytic leukaemia (APL) is characterized by the presence of the t(15;17) chromosomal translocation that generates the *PML-RARA* fusion gene [1]. *PML-RARA*, formed by the fusion of the promyelocytic leukaemia (*PML*) and retinoic acid receptor α (*RARA*) genes, is a constitutively active nuclear receptor that exerts transcriptional repression of *RARα* target genes leading to enhanced self-renewal capacity and myeloid differentiation block [2]. In approximately half of all cases, APL cells harbor additional mutations in genes such as *FLT3*, *WT1*, *NRAS* or *KRAS* [3, 4]. Mice expressing *PML-RARA* under the control of various promoters develop APL-like disease with variable penetrance and only after a significant period of latency (6–16 months) [5, 6]. These observations suggest a long latency between *PML-RARA* acquisition and APL onset, as reported for somatic mutations associated with acute myeloid leukaemia with a normal karyotype (AML-NK) [7]. Here, to investigate whether *PML-RARA* is detectable in the blood before APL onset, we study four APL cases with blood DNA available, from the same individual, 2–12 years before diagnosis.

Four individuals with paired APL and pre-leukaemic blood DNA samples were identified (Fig. 1a). APL DNA samples were shallow whole-genome sequenced on Illumina HiSeq using a 150 bp paired-end protocol preceded by DNA sonication to generate fragments of 450 nucleotides (nt) average length. FASTQ files were aligned to DNA sequences of the *PML* and *RARA* genes. Aligned reads were analyzed using RNAmut v1.0 [8] and this directly identified the precise location of 3/4 patient-specific breakpoints (by reads crossing the breakpoint), whilst the location of the fourth breakpoint was narrowed to a 20 nt window (Fig. 1b). Patient-specific primer pairs were then designed to amplify across each breakpoint using the polymerase chain reaction (PCR) (Fig. 1c). To determine the sensitivity for detecting each breakpoint by PCR, leukaemic DNA samples were suspended at 20 ng/μL and iteratively diluted tenfold to a dilution of 1:10,000 into equimolar (20 ng/μL) pooled normal blood DNA from 10 healthy subjects. All 4 primer pairs specifically amplified their cognate patient-specific breakpoints (Fig. 1d). Serial dilutions demonstrated sensitivity down to a 1:1000 dilution of cognate APL DNA (equivalent to 200 pg/μL of DNA). Despite this high sensitivity, none of the *PML-RARA* fusions was identified in their relevant pre-leukaemic samples (Fig. 1d, representative of triplicate PCRs).

**Fig. 1:**
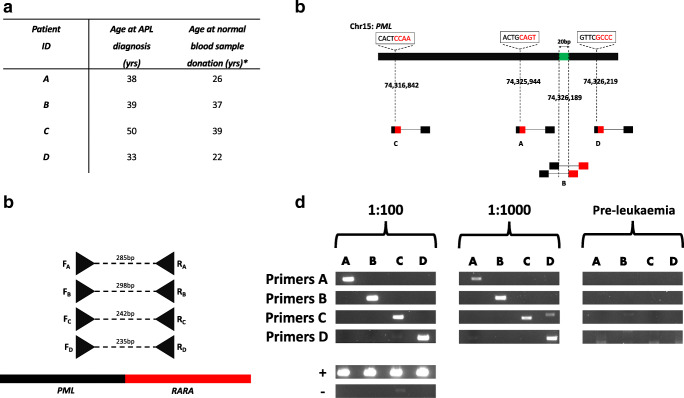
PML-RARA is not detectable in blood DNA 2–10 years prior to the diagnosis of APL. **a** Demographics of patients studied. *Samples were collected for the purposes of registering as blood stem cell donors at the Karaiskakio foundation. **b** Identification of chimeric reads crossing the *PML-RARA* breakpoints in 4 cases of APL (patients A–D). The genomic loci of each breakpoint are highlighted with respect to their position on the PML gene (black) of chromosome 15. Reads mapping to *RARA* gene (chromosome 17) are highlighted in red. For individual B, the precise breakpoint was not located, but narrowed to a 20 bp window (green). **c** Patient-specific forward (F) and reverse (R) primers were designed to amplify each *PML-RARA* breakpoint. **d** Gel electrophoresis of diluted leukaemia (left and central columns) and neat pre-leukaemia (right column) DNAs following PCR with patient-specific primer pairs. PML primers were used as positive controls (+) and neat normal DNA with patient-specific primers as negative controls (-)

In summary, our study of paired DNA samples suggests that the pre-clinical clonal history of APL may not be as long as that of AML-NK, where mutation-bearing clonal hematopoiesis (CH) clones can be detected years in advance of their progression to frank leukaemia^11^. However, with the exception of *JAK2* V617F, CH is almost entirely driven by mutations in epigenetic (DNMT3A, TET2, ASXL1, IDH1, IDH2), splicing (SF3B1, SRSF2, U2AF1) or apoptosis-related (TP53, PPM1D) genes [9, 10]. By contrast, bona-fide leukaemogenic mutations in genes such as *NPM1*, *CEBPA* and *MLL* have not been identified prior to the diagnosis of AML-NK, reflecting what we have observed for *PML-RARA*. Collectively these observations suggest that acquisition of such mutations leads to the development of AML after a relatively short time. Alternatively, mutation-bearing cells may not reach the circulation as a consequence of the effects of these mutations in blocking myeloid differentiation [6]. In either case, our findings suggest that, unlike AML-NK [7], APL does not commonly have a long pre-clinical phase that can be detected in the DNA of circulating blood cells.

## References

[1] de Thé H, Lavau C, Marchio A, Chomienne C, Degos L, Dejean A (1991). The PML-RAR alpha fusion mRNA generated by the t(15;17) translocation in acute promyelocytic leukemia encodes a functionally altered RAR. Cell..

[2] Wojiski S, Guibal FC, Kindler T, Lee BH, Jesneck JL, Fabian A, Tenen DG, Gilliland DG (2009). PML-RARalpha initiates leukemia by conferring properties of self-renewal to committed promyelocytic progenitors. Leukemia..

[3] Papaemmanuil E, Gerstung M, Bullinger L, Gaidzik VI, Paschka P, Roberts ND, Potter NE, Heuser M, Thol F, Bolli N, Gundem G, van Loo P, Martincorena I, Ganly P, Mudie L, McLaren S, O’Meara S, Raine K, Jones DR, Teague JW, Butler AP, Greaves MF, Ganser A, Döhner K, Schlenk RF, Döhner H, Campbell PJ (2016). Genomic classification and prognosis in acute myeloid leukemia. N Engl J Med.

[4] Madan V, Shyamsunder P, Han L (2016). Comprehensive mutational analysis of primary and relapse acute promyelocytic leukemia. Leukemia.

[5] Westervelt P, Lane AA, Pollock JL, Oldfather K, Holt MS, Zimonjic DB, Popescu NC, DiPersio JF, Ley TJ (2003). High-penetrance mouse model of acute promyelocytic leukemia with very low levels of PML-RARalpha expression. Blood..

[6] Grisolano JL, Wesselschmidt RL, Pelicci PG, Ley TJ (1997). Altered myeloid development and acute leukemia in transgenic mice expressing PML-RAR alpha under control of cathepsin G regulatory sequences. Blood..

[7] Abelson S, Collord G, Ng SWK (2018). Prediction of acute myeloid leukaemia risk in healthy individuals. Nature.

[8] Gu M, Zwiebel M, Ong SH, Boughton N, Nomdedeu J, Basheer F, Nannya Y, Quiros PM, Ogawa S, Cazzola M, Rad R, Butler AP, Vijayabaskar MS, Vassiliou GS (2019). RNAmut: robust identification of somatic mutations in acute myeloid leukemia using RNA-seq. Haematologica..

[9] Xie M, Lu C, Wang J, McLellan MD, Johnson KJ, Wendl MC, McMichael JF, Schmidt HK, Yellapantula V, Miller CA, Ozenberger BA, Welch JS, Link DC, Walter MJ, Mardis ER, Dipersio JF, Chen F, Wilson RK, Ley TJ, Ding L (2014). Age-related mutations associated with clonal hematopoietic expansion and malignancies. Nat Med.

[10] Jaiswal S, Fontanillas P, Flannick J, Manning A, Grauman PV, Mar BG, Lindsley RC, Mermel CH, Burtt N, Chavez A, Higgins JM, Moltchanov V, Kuo FC, Kluk MJ, Henderson B, Kinnunen L, Koistinen HA, Ladenvall C, Getz G, Correa A, Banahan BF, Gabriel S, Kathiresan S, Stringham HM, McCarthy MI, Boehnke M, Tuomilehto J, Haiman C, Groop L, Atzmon G, Wilson JG, Neuberg D, Altshuler D, Ebert BL (2014). Age-related clonal hematopoiesis associated with adverse outcomes. N Engl J Med.

